# Telomeres and Telomerase in Hematopoietic Dysfunction: Prognostic Implications and Pharmacological Interventions

**DOI:** 10.3390/ijms18112267

**Published:** 2017-10-28

**Authors:** Theresa Vasko, Andrea Kaifie, Matthias B. Stope, Thomas Kraus, Patrick Ziegler

**Affiliations:** 1 Institute for Occupational and Social Medicine, RWTH Aachen University, 52074 Aachen, Germany; tvasko@ukaachen.de (T.V.); akaifie@ukaachen.de (A.K.); tkraus@ukaachen.de (T.K.); 2 Department of Urology, University Medicine Greifswald, 17475 Greifswald, Germany; stopem@uni-greifswald.de

**Keywords:** telomere, telomerase, hematologic dysfunction

## Abstract

Leukocyte telomere length (TL) has been suggested as a marker of biological age in healthy individuals, but can also reflect inherited and acquired hematopoietic dysfunctions or indicate an increased turnover of the hematopoietic stem and progenitor cell compartment. In addition, TL is able to predict the response rate of tyrosine kinase inhibitor therapy in chronic myeloid leukemia (CML), indicates clinical outcomes in chronic lymphocytic leukemia (CLL), and can be used as screening tool for genetic sequencing of selected genes in patients with inherited bone marrow failure syndromes (BMFS). In tumor cells and clonal hematopoietic disorders, telomeres are continuously stabilized by reactivation of telomerase, which can selectively be targeted by telomerase-specific therapy. The use of the telomerase inhibitor Imetelstat in patients with essential thrombocythmia or myelofibrosis as well as the use of dendritic cell-based telomerase vaccination in AML patients with complete remissions are promising examples for anti-telomerase targeted strategies in hematologic malignancies. In contrast, the elevation in telomerase levels through treatment with androgens has become an exciting clinical intervention for patients with BMFS. Here, we review recent developments, which highlight the impact of telomeres and telomerase targeted therapies in hematologic dysfunctions.

## 1. Introduction

Telomeres consist of hundreds to thousands of repeated double strand hexanucleotides (in humans: (TTAGGG)n), a single-stranded overhang, as well as specific proteins that coat them (telosome or shelterin protein complex) [1,2]. Telomeres are simple but essential DNA sequences whose loss does not impact on the transmission of genetic information. The main function of telomeres is to form a stable and recognized end of the chromosome that avoids it being perceived from the cell as a naked chromosome or a DNA fragment that needs to be repaired [3]. Chromosome ends, uncapped by telomeres, are sensitive to degradation or fusion and can activate a DNA damage checkpoint response [4].

In vertebrate cells, each cell division in vitro and in vivo is associated with the loss of 50–200 base pairs (bp) of telomeric TTAGGG repeats due to the “end-replication” problem [5]. As a consequence, telomere length (TL) both reflects and limits the replicative life-span of somatic cells and functions as a “mitotic clock” [1]. When cells get critically short telomeres, replicative senescence precludes them from end to end fusions, enzymatic degradation, or nonreciprocal translocations that eventually result in chromosomal instability, aneuploidy, and tumorigenesis [6]. However, cells with malignant potential are able to escape replicative senescence by failure or suppression of the DNA damage response: for example, the loss of the tumor protein 53 and the activation of telomere maintenance mechanisms [7]. As a result, these cells continue to grow, leading to immortalization and frank malignancy. A majority of human cancers exhibit critically short telomeres, suggesting that tumors indeed can arise from genetically instable cells. Real evidence for a contribution of telomere crisis and dysfunction to cancer progression can be found in chronic lymphocytic leukemia (CLL) [8] and myelodysplastic syndrome (MDS) [9], where the occurrence of critical short telomeres correlates with the presence of large-scale genomic rearrangements (CLL) and DNA damage (MDS), which are not detectable in samples with longer telomeres.

Telomere shortening can be counteracted by telomerase, the telomere extending enzyme, which is embedded into the shelterin protein complex [10]. Telomerase consists of two essential components: TERT (reverse transcriptase component) and TERC (functional or template RNA that recognizes the telomeres at the end of chromosomes) [10]. While TERC can be found in abundance in tumor and non-tumor cells, the catalytic subunit TERT is expressed in cells with self-renewal activity such as hematopoietic stem and progenitor cells (HSPCs), proliferating lymphocytes and the regenerative basal layer of the epidermis in human skin [11]. In addition, germline and embryonic stem cells express telomerase [12]. Ectopic expression of telomerase reverse transcriptase in human (hTERT) leads to cellular immortalization and bypass of replicative senescence. Furthermore, continued expression of hTERT is necessary to maintain immortality and 90% of all tumor cells express hTERT at high levels [13]. Telomeres in hTERT positive cells are continuously stabilized below known crisis thresholds, allowing these cells to keep multiplying and to avoid ageing. Although it is known that cancer cells continue to grow because of reactivation of dormant hTERT, the genetic alterations that activate hTERT during neoplastic transformation have remained a mystery for a long time. However, recent findings suggest that recurrent, cancer specific mutations in its gene regulatory region activate hTERT during neoplastic transformation [14]. For instance, it was found in melanoma, that within the hTERT promoter, a single base pair change creates a new binding site for the E26 transformation-specific transcription factor (ETS). This was further shown to be the key point in turning on hTERT expression and that 80% of all melanoma cases have this base pair change [14]. In addition, in urothelial cancer (UC)-derived cell lines, hTERT promoter mutations (−124 C→T; −146 C→T and a −57 A→C mutation) correlated with higher telomerase expression levels and high telomerase activity [15]), equally suggesting promoter mutations as a possible cause for hTERT reactivation.

However, hTERT expression in health and disease is a very delicate balance, and individuals with short telomeres due to failure in telomere maintenance mechanisms (telomeropathies) are prone to genomic instability and have an increased risk for organ failure. Telomeropathies, such as dyskeratosis congenita, can manifest in the BM (as bone marrow failure syndromes, BMFS), liver, and the lung, and their link to short telomeres can either be related to inherited genetic mutations or simply to an acquired pathophysiology of the disease. Androgenic anabolic steroids, which have been used to improve blood counts of patients with BMFS since the 1960s, concurrently slow telomere shortening or even sustain telomere elongation in leukocytes most likely by an elevation in telomerase expression levels in the underlying HSPC compartment [16]. These anabolic effects of androgenic steroids on TL are significant and clinically relevant and provide a promising approach for “crisis intervention” in other telomere maintenance disorders [17].

## 2. Prognostic and Predictive Implications of Telomere Length (TL) Measurements

In general, TL of peripheral blood cells decreases with age and is subject to considerable, genetically determined variation at any given age. When measured by quantitative fluorescence in situ and flow cytometry (flow-FISH)-based methodologies [18], which have emerged as the clinical standard for diagnosing telomere syndromes [19], the TL of leukocytes and lymphocytes can be independently assessed in the same sample. As leukocytes are postmitotic cells and their TL reflects the TL of the hematopoietic stem cells (HSCs) they are derived from [20] the estimation of average TL of blood leucocytes allows to gain insights into division patterns of HSCs in an in vivo situation. Normal HSCs which are set for maximum expansion during early life, decrease TL logarithmically during adolescence and slow down to linear kinetics in adults [20]. Furthermore, whereas the loss of telomeric repeats declines with age in leukocytes, it slightly accelerates in lymphocytes [21]. Leukocyte TL has therefore been suggested as a marker of biological age and geriatric assessment in normal individuals [1] but can also reflect inherited and acquired hematopoietic dysfunctions or indicate an increased turnover of the HSPC compartment. In addition, TL is able to predict the therapy response in chronic myeloid leukemia (CML) and in BMFS, for instance. Furthermore, telomere measurement can be used as a clinically useful screening tool for consecutive genetic sequencing of selected genes encoding proteins within the shelterin complex in patients with inherited BMFS to detect mutations that lead to a reduced telomerase activity. Impact of TL and telomerase measurement on the prognosis of hematologic disorders are summarized in Table 1.

## 3. Chronic Myeloid Leukemia (CML)

Chronic myeloid leukemia (CML) is a clonal, myeloproliferative disorder that originates in the hematopoietic stem and progenitor compartment (HSPC) and that leads to a marked expansion of myeloid cells [56]. CML originates from a reciprocal translocation between one chromosome 9 and one chromosome 22 t(9;22), generating the Philadelphia chromosome (Ph) and creating the *BCR-ABL* oncogene [57]. In CML, the telomeres of leukocytes are significantly shorter as compared to telomeres of normal lymphocytes of the same individual as well as telomeres of leukocytes of a healthy control group [22]. Leukocytes from patients in accelerated phase or blast phase (AP/BP) show significantly shorter average TL than cells from patients in chronic phase or cytogenetic remission [23]. This leads to the conclusion, that in CML increased cellular turnover of clonal BCR-ABL positive HSPCs causes significantly shortened telomeres in peripheral blood myeloid cells [22]. These findings suggest, that the extend of telomere shortening in CML correlates with disease stage and that the telomeres in the neoplastic clone eventually become critically short and may drive progression of chronic phase CML to accelerated phase or blast crisis [22].

During tyrosine kinase inhibitor (TKI) treatment, patients with Imatinib treatment duration > 144 days showed significantly longer telomeres in comparison to patients who recently started TKI treatment up to day 144 of treatment. Median telomere length in major remission was found to be significantly longer compared to patients without response to treatment measured either by cytogenetics, interphase FISH or quantitative RT-PCR [24]. In conclusion, the increase in TL under treatment with TKI reflects a shift from Ph+ to Ph− cells in patients with CML, thereby reflecting response to TKI treatment and the comeback of normal (BCR-ABL negative) polyclonal hematopoiesis [24]. These findings further emphasize that in CML patients, BCR-ABL negative hematopoiesis is sustained by non-neoplastic HSPCs and argues against a substantial mean TL deficit in the normal, Ph− hematopoietic stem cell compartment.

In addition, it has been found, that in CML, TL analysis could indicate the severity of the disease and thus may provide important information for treatment decisions: when TL of newly diagnosed CML patients is evaluated before treatment, patients with the longest telomeres show a lower clinical risk profile in comparison to patients with shorter telomeres and may predict response to initial TKI therapy after 12 and 18 months according to ELN criteria [25]. These data further suggest that CML chronic phase (CP) can be classified into early and late CP, depending on the degree of expansion of the leukemic clone in the underlying HSPC compartment.

Telomerase activity is upregulated in CP-CML as compared to HSPCs from normal individuals and increases up to 50-fold in blastic phase CML. Telomerase activity therefore correlates with acute versus chronic forms of CML and disease progression [58,59]. In addition, high levels of telomerase in leukemic progenitors at baseline may be a feature of both the malignant phenotype and rapid cycling.

## 4. Chronic Lymphocytic Leukemia (CLL)

Chronic lymphocytic leukemia is characterized by the clonal expansion of abnormal lymphocytes (B cells) in the peripheral blood, which is in part dependent on several genetic and epigenetic alterations, B-cell receptor signaling, and the interaction between CLL cells and the tissue microenvironment [60,61]. TL measurement is of prognostic value in CLL. In a recent meta-analysis, Adam and colleagues reported a consistent association of longer telomeres measured by quantitative PCR and longer overall survival in CLL. Long (versus short) telomeres improved clinical outcomes and including data from 2357 patients, the pooled hazard ratio (HR) for overall survival in CLL was 0.45 (95% CI= 0.29–0.71, *p* < 0.001) [26]. In addition, in CLL, TL predicts progression of the disease and time to first treatment in multivariate analyses of models incorporating established biomarkers, including mutational status of immunoglobulin variable heavy-chain genes (*V_H_*) [27]. *V_H_* unmutated cells have shorter telomeres than *V_H_* mutated cells, and longer telomeres are associated with increasing V_H_ mutational load. TL therefore appears to be a very good surrogate marker for the IGHV mutational status and CLL patients with long telomeres and mutated IGVH had a significantly better prognosis than patients with short telomeres and unmutated IGVH [27]. Furthermore, TL negatively correlates with the expression of ZAP-70 and CD38 [28], and patients carrying the shortest telomeres at time of diagnosis have a higher risk profile, including the occurrence of prognostically unfavorable high risk genomic aberrations including deletions of −11q or −17p [29]. In many malignancies, it has not been formally proven that a high turnover of the neoplastic clone and the resulting short telomeres favor the acquisition of genetic instability, or if complex chromosomal aberrations lead to a higher cellular turnover in the first place. Lin et al. provided convincing evidence that in CLL, disease-initiating cells undergo a telomere-based crisis which drives genomic instability and facilitate disease progression [8].

Telomerase expression and activity have both been investigated as biomarker for disease progression in CLL [62,63]. The *hTERT* transcript contains alternate splicing sites, and some mRNA variants of *hTERT* prevent the expression of a catalytically active (full length) telomerase with the possible result, that telomeric ends are not longer maintained [64]. In a study where 59% of CLL samples had no detectable telomerase activity as measured by polymerase chain reaction (PCR)-based telomeric repeat amplification protocol (TRAP) assay, no correlation between telomerase activity and overall survival could be detected [63]. However, others report a prognostic value of hTERT expression in a series of 134 B-CLL cases [62] Quantifying either all hTERT transcripts (AT) or only the full length (FL) transcript encoding the functional protein, they report an inverse correlation between the expression of FL telomerase and the percentage of IGVH mutational load. Using a cutoff level of 40 copies of FL transcripts, IGVH unmutated cases with low hTERT levels had an overall survival close to mutated cases with high hTERT levels [62]. Whereas in the first case [63] the lack of impact of telomerase activity on the patient survival supports the concept that CLL is a disease of mature cells whose principal characteristic is inability to die (resistance to apoptosis) rather than to over-proliferate [65], the second report postulates that the hTERT transcript level could serve as a prognostic indicator for overall survival in B-CLL patients [62].

## 5. Myelodysplastic Syndrome (MDS)

Myelodysplastic syndromes (MDS) are a heterogenous group of clonal diseases in HSPCs, which result in ineffective hematopoiesis and subsequent cytopenia and dysplasia [66]. Patients with MDS have been shown to have short TL compared to control individuals [30,67,68], and in a recent mouse model of progressive telomere shortening, telomere dysfunction lead to persistent physiological DNA damage and classical MDS features, strongly demonstrating the contribution of telomeres to the pathophysiology of MDS and malignant transformation [9]. Various karyotopic subgroups demonstrate different TL profiles in MDS, and further changes in karyotype, which usually develop under involvement of telomere shortening are part of the course of MDS and mostly linked to a progress of the disease and leukemic transformation in AML. Therefore, prospective monitoring of TL in MDS could be of clinical importance to the early detection of disease progression, and assessment of TL is of relevant value as a possible marker for patients more at risk [31]. In general the significance of not only the mean TL, but in particular, single critically short telomeres have been shown to cause chromosomal instability [69,70]. Therefore, some studies have analyzed TL at the single cell level in MDS patients combining chromosome banding and fluorescence in-situ hybridization techniques (FISH) [71]. However, these studies were not able to detect differences in TL between normal and aberrant metaphases in cells from patients with MDS. Therefore, the relevance of single critically short telomeres for the generation of chromosomal instability in development of MDS remains to be determined.

In a recent study [72], no significant correlation between telomerase activity and TL could be found in MDS patients (*n* = 53), and for overall survival, there was no significant difference between high and low telomerase activity according to the log rank method. However, patients with high telomerase activity showed a significantly shorter interval for transformation to acute leukemia (*p* = 0.010), suggesting high telomerase activity as a poor prog­nostic marker in MDS.

## 6. Bone Marrow Failure Syndromes

Bone marrow failure syndromes (BMSF) are a heterogenous set of potentially fatal diseases that can develop at any stage of life. They are characterized by peripheral cytopenia in at least two of the three blood lineages and marrow hypoplasia. A subpopulation of patients with BMFS are typically characterized by ultra-short telomere length (USTL, i.e., beyond the 5% percentile of normal individuals) due to altered telomere maintenance mechanism (telomeropathy) [33]. Telomeropathies, such as Dyskeratosis congenita (DKC), can manifest in the BM, liver, and the lung, and their link with short telomeres can either be related to genetic mutations in hTERT or in genes encoding proteins of the shelterin complex including TRF1, TRF2, TIN2, POT1, TPP1, and RAP1 (Genes Dev. 2005;19(18):2100) associated factors such as dyskerin (DKC1) [34], hTERC [35], TINF2, NOP10 and NHP2 (inherited) or simply to the pathophysiology of the disease (acquired) [33,36]. TL measurement in these patients therefore presents a clinically useful screening tool for consecutive genetic sequencing of selected genes encoding proteins and associated factors within the shelterin complex.

In contrast to the constitutional bone marrow failure observed in DKC patients, in acquired aplastic anemia (AA), HSPCs are destroyed by the patient’s own immune system [37]. In order to compensate for stem cell loss and low blood cell counts [38] this quantitative defect leads to a limited replicative capacity of the remaining HSPC pool and results in the penetrance of the short telomere phenotype in peripheral blood cells. Telomere shortening in leukocytes of patients with aplastic anemia correlates with the degree of (pan-)cytopenia observed [73]. However, after immunosuppressive therapy, TL was found to be restored, while untreated patients and non-responders with persistent severe pancytopenia showed marked and significant telomere shortening. These results therefore suggest an extensive proliferation of HSC in subgroups of AA patients [39].

Eventually, telomeres become critically short in cells with malignant potential. In the absence of mechanisms such as p53 signaling of DNA damage or activation of alternative modes of telomere maintenance, cells with critically short telomeres can continue to replicate and expand on a clonal level. Therefore, all patients with telomeropathies are prone to genomic instability and have an increased risk for cancer [37]. For example, in severe AA, clonal evolution to acute leukemia (AML) is a major adverse event [37], and the major risk factor for malignant clonal evolution, mainly monosomy 7, is having very short telomeres [40]. In a cohort of patients with severe aplastic anemia, patients within the lowest quartile of TL had a about five- to seven-fold higher rate of evolution to monosomy 7 than patients who had longer telomeres (95% CI, 8.7–37.5%) [40]. This suggests that patients that undergo clonal evolution have an extraordinary accelerated telomere attrition and can be identified by telomere measurement.

## 7. Telomerase

Because of an inability to completely replicate their telomeres, chromosomes progressively shorten during each replication cycle. Telomere shortening is counteracted by the enzyme telomerase, which adds telomeric nucleotide sequences to the end of the chromosomes [74]. Telomerase exists as a ribonucleotide protein complex, consisting of an RNA component (Telomerase RNA component: TERC), a catalytic subunit and multiple associated proteins. Reverse transcriptases used by retroviruses generate single-stranded cDNA complementary to the single-stranded genomic RNA template. Telomerase utilizes a precisely defined internal RNA template, except for telomerase without internal RNA template identified in mitochondria [75]. The gene coding for human TERC maps to the distal end of chromosome 3q [76] and consists of 451 nucleotides, including an 11 bp long RNA template (5′-CUAACCCUAAC-3′) complementary to human telomere repeats (TTAGGG)n [76]. During telomere elongation, the 5′-end of the RNA template stably associates with the catalytic subunit of the telomerase enzyme while free nucleotides of its 3′-end align to the free 3′-end of the chromosome by Watson Crick base pairing [77]. Telomerase then polymerizes telomere repeats onto the DNA, extending the length of the guanine-rich (G-rich) single-stranded overhang. The telomeric G-rich strand runs in the 5′ to 3′ direction towards the termini of the template region. After translocation of the telomerase enzyme, a new elongation steps starts. Telomerase is only capable of synthesizing the G-rich strand. The cytosine-rich (C-rich) strand is filled in by conventional DNA polymerases [78].

The catalytic subunit of human telomerase is a single copy gene that maps to chromosome 5p15.33, remarkably close to the telomere at the extreme terminus of chromosome 5p [79]. Telomerase is synthesized as a 127 kDa protein with specific reverse transcriptase motifs at the c-terminus [80] and a so called T-motif, which contributes to the binding of the RNA template [81]. While the RNA subunit of telomerase can be found in abundance in tumor and non-tumor cells, the catalytic subunit is expressed in 90% of all tumors at high levels [12]. Telomeres in these cells are therefore continuously replenished by telomerase as the cells keep dividing.

## 8. Telomerase Inhibition

With the reactivation of hTERT expression, cancer cells have developed a basic difference compared to their precursors, which makes them one possible key to selectively target cancer cells by pharmacological approaches. Telomerase specific therapy is a very promising area of research and very effective when used in combination with other cancer therapies [82]. By reducing the number of TTAGGG repeats of successive generations, telomerase inhibitors induce senescence or apoptosis of affected cells, thereby limiting the growth of tumors [83,84,85]. However, as cells only lose 50–200 base pairs of telomeres per cell division, a long time lag between onset of therapy and treatment response may prevent telomerase inhibitors from being an effective anti-tumor strategy on their own [82]. Indeed, studies with telomerase inhibitors are frequently discontinued in patients with solid tumors, because interim analyses of the clinical data show no survival benefit from the drugs. However, this might be different in chronic disordes such as Philadelphia-Chromosome negative myeloproliferative neoplasias (Ph-MPN). MPN, including polycythemia vera, essential thrombocythemia, and primary myelofibrosis are clonal diseases of hematopoietic progenitor cells associated with common mutations in the Janus kinase 2 (*JAK2*) gene, the gene encoding the thrombopoietin receptor (*MPL*), and the calreticulin (*CALR*) gene [48,49,50,51,52]. TL is reduced and telomerase activity is upregulated in bone marrow derived hemopoietic progenitor cells from Ph-MPN patients providing a rationale for the use of telomerase inhibitors in the treatment of MPN [53].

Two phase II clinical trials reported a clinical response in MPN patients treated with the telomerase inhibitor GRN163L (Imetelstat) [54,55]. Imetelstat is an oligonucleotide which does not work by an antisense mechanism, but inhibits enzymatic activity of telomerase by binding with high affinity and specificity to the intrinsic RNA template thereby blocking its association with the catalytic subunit [86]. In patients suffering from essential thrombocythemia, characterized by an excess of platelets, who did not show a response to prior therapies or had unacceptable side effects, Imetelstat treatment demonstrated a hematological and molecular response [54]. A total of 18 patients were treated with 7.5 or 9.5 milligrams of Imetelstat per kg of bodyweight once a week, until platelet counts of approximately 250 to 300 per cubic millimeter were reached. All 18 patients showed a hematologic response, while 16 patients reached a complete hematologic response. Seven out of the 18 patients were positive for the *JAK2* mutation and showed a molecular response by reduction in *JAK2* V617F mutant alleles. Furthermore, patients with calreticulin (CALR) and MPL mutations showed a reduced mutant allele burden, thereby providing evidence of an anticlonal activity of Imetelstat [54]. Similarly, a study enrolling patients with myelofibrosis who have relapsed or were refractory to a JAK inhibitor, reported complete and partial remissions, including reversal of bone marrow fibrosis and molecular responses, but only in seven out of 33 patients (21%) [55]. Side effects of Imetelstat included cytopenias, which showed the main dose limiting toxicity, but appeared to be manageable with dose modification and retreatment guidelines. The other most commonly reported adverse events included fatigue and gastrointestinal symptoms. Patients in these studies also experienced elevated liver enzymes, which resolved to normal or baseline in the majority of patients after Imetelstat was withdrawn [54]. However, TL did not predict the treatment response, and in the myelofibrosis cohort, TL did not change during treatment. Telomerase inhibition therefore seems not to be the only mode of action exerted by Imetelstat in MPN, and some of the responses could also be due to off-target effects of the drug, such as myelosuppression [87].

In addition to Imetelstat, immunotherapy tries to exploit the high expression of telomerase in cancer cells. Spontaneous, antileukemic, cytotoxic T cells against telomerase-derived antigens have been shown to occur naturally in B-cell chronic lymphocytic leukemia [88] giving a rationale for a telomerase vaccination strategy to direct an immune mediated cell kill against telomerase. In addition, telomerase-based vaccination has been safely applied in patients with solid tumors and has been shown to induce functional antitumor CD8 T-lymphocytes [89]. AST-VAC1 (formerly known as GRNVAC1) is an autologous telomerase-based dendritic cell vaccine which has successfully completed a phase 2 trial in patients with acute myeloid leukemia (AML) in complete remission (CR) [90]. The vaccination with AST-VAC1 involves autologous dendritic cells transfected ex vivo with mRNA encoding telomerase amino acids 168 through 1132 and a portion of lysosomal associated membrane protein 1 (LAMP) before reimplantation into patients [91,92]. Thirty-three patients with first or second complete remission (CR) and with intermediate-risk or high-risk cytogenetics were enrolled in the study. Twenty-one patients received more than three doses the vaccination, 19 of whom were in CR. Fourteen patients remained free of disease recurrence, while nine out of these 14 patients developed telomerase-specific T-cell responses. In the long-term follow-up, seven patients remained free of disease with telomerase-specific T-cell responses [90]. Telomerase-directed therapy was well tolerated with no severe toxicities reported, with the exception of one patient who developed idiopathic thrombocytopenic purpura. The recurrence free survival reported in this study was favourable compared to historical controls, and upon further confirmation in additional clinical trials, dendritic cell based telomerase vaccination might be a promising immunotherapeutic strategy for AML patients in CR [90].

## 9. Induction of Telomerase Expression

Shortened telomeres are a characteristic BMSF and can be due to heterozygous hTERT mutations, which compromise telomerase function by haploinsufficiency [93]. Androgenic hormones have been useful in treating bone marrow failure syndromes since the sixties, but the underlying mechanism for treatment success in some cases but not in others has remained unknown. Interestingly, telomerase catalytic activity has been shown to be responsive to stimulation by sex hormones via upregulation of hTERT mRNA [41]: hematopoietic cells from patients carrying mutations within the telomerase complex responded to androgen treatment with the upregulation of hTERT in vitro [42] and in mice with aplastic anemia, induced by short telomeres, testosterone therapy resulted in telomerase up-regulation and increase in telomere length [43]. Danazol [44] is a synthetic hormone that reduces levels of circulating estradiol in both men and women because of inhibited secretion of pituitary gonadotropins and reductions in gonadal steroidogenesis [45]. In a phase ½ trial, 27 patients with shortened telomeres (below 1% percentile of age adjusted control) and pancytopenia were given 800 mg of Danazol daily for 24 months [46]. Twenty-one patients had mutations in genes and associated factors of the shelterin complex including hTERT (*n* = 10), TERC (*n* = 7), DKC (*n* = 3) and RTEL (*n* = 1). The primary endpoint of the study was a 20% reduction in the rate of telomere attrition at 24 months. The study was stopped early after the first twelve patients all met the primary endpoint. All 27 patients were then assessed and unexpectedly almost all patients had a gain in telomere length with a mean increase of 386 base pairs [46]. Hematologic responses also occurred in 10 of the 12 patients who were followed for 24 months including increases in hemoglobin, neutrophils, and platelets usually leading to independence from transfusion. Known adverse effects of Danazol were frequent but low grade and included elevated liver enzymes and muscle cramps. The authors concluded that pharmalogical preservation or modest elongation of leukocyte TL in patients with very short telomeres was achieved and was associated with an improvement in blood counts [46]. Although the mechanisms of action of Danazol, which is a structurally nonaromatizable androgen [45], have not been fully elucidated, this and other studies [16] provide evidence for sustained telomere elongation in HSPCs induced by a pharmacological approach in vivo. Screening of TL in patients with BMFS might therefore not only be a prognostic marker, but also a predictive tool for response to treatment in patients receiving androgen derivatives.

## 10. Future Directions

With the confirmation of TL as an independent prognostic factor for overall survival in a recent meta-analysis, the concept of TL measurements in CLL has been proven to provide clinically relevant information. However, due the lack of standardization and the variability of methods to measure TL, the outcome of TL on other advanced myeloid cancers is not clear. Here, agreements are necessary that define the method of choice for measurements and its standardization.

With the proof-of-concept data of Imetelstat in patients with ET and myelofibrosis, and the use of androgenic hormones to increase telomerase expression in patients with BMFS, telomerase targeted therapies provide important clinical interventions for hematological dysfunctions. In both cases, further randomized clinical studies are necessary to assess drug benefit-risks, determine the most effective dosing schedules and to clarify the mechanism of action.

## Figures and Tables

**Table 1:** Summary of evidence between telomere length and hematological diseases.

Evidence	(Hematological) Diseases	Reference
telomere length	Chronic myeloid leukemia (CML) significantly shorter telomeres of leukocytes in accelerated or blast phase compared to chronic phase or cytogenetic remission increased cellular turnover of clonal BCR-ABL positive HSPCs→significantly shortened telomeres in peripheral blood myeloid cells response to tyrosine kinase inhibitor (TKI) treatment	[22,23,24,25]
	Chronic lymphocytic leukemia (CLL) disease initiating cells undergo telomere crisis→genomic instability, disease progression Telomere length (TL) predicts disease progression/time to first treatment longer telomeres predict longer overall survival TL as surrogate marker for mutational status of IGVH TL negatively correlates with ZAP-70/CD38 expression patients with shortest telomeres have higher risk for genomic aberrations	[8,26,27,28,29]
	Myelodysplastic syndrome (MDS) progressive telomere shortening → DNA damage TL as possible marker for patients with higher risk	[9,30,31,32]
	Bone marrow failure syndromes (BMFS) genetic mutations in hTERT or proteins of shelterin complex → telomeropathy, e.g., Dyskeratosis congenita → ultra-short telomere length increased risk for cancer response to androgenic hormones, e.g., Danazol autoimmune reaction against HSPCs in acquired aplastic anemia (AA)→compensation of stem cell loss→limited replicative capacity of remaining HSPC pool→short telomeres in peripheral blood cells response to immunosuppressive therapy extraordinary accelerated telomere attrition predicts evolution to acute myeloid leukemia (AML)	[33,34,35,36,37,38,39,40,41,42,43,44,45,46]
hTERT promoter mutations	Melanoma 80% of all cases: enhanced telomerase expression levels due to single base pair change in hTERT promoter region	[14,47]
	Urothelial cancer (UC) derived cell lines higher telomerase expression levels and high telomerase activity due to hTERT promoter mutation	[15]
telomerase activity	Philadelphia-Chromosome negative myeloproliferative neoplasias (PH-MPN) reduced TL upregulated telomerase activity treatment with telomerase inhibitors, e.g., Imetelstat	[48,49,50,51,52,53,54,55]
